# Association between long-term PM_2.5_ exposure and risk of urticaria and allergic rhinitis in Korean children: a nationwide longitudinal cohort study

**DOI:** 10.4178/epih.e2026020

**Published:** 2026-05-07

**Authors:** Young A Park, Hyemin Jang, Whanhee Lee, Jongmin Oh, Jung Won Lee, Hae Soon Kim, Jung Eun Choi, Rosie Lee, AiMS-CREATE, Ji Hyen Lee, Eun-Hee Ha

**Affiliations:** 1 Institute of Ewha–Seoul Clinical Laboratories for Environmental Health (IESEH), Ewha Womans University College of Medicine, Seoul, Korea; 2 Department of Pediatrics, Ewha Womans University College of Medicine, Seoul, Korea; 3 Department of Public Health Sciences, Graduate School of Public Health, Seoul National University, Seoul, Korea; 4 School of Biomedical Convergence Engineering, Pusan National University, Yangsan, Korea; 5 Department of Occupational and Environmental Medicine, Ewha Womans University College of Medicine, Seoul, Korea; 6 Graduate Program in System Health Science and Engineering, Ewha Womans University, Ewha Medical Research Institute, College of Medicine, Seoul, Korea

**Keywords:** Child, Adolescent, Particulate matter, Air pollutants, Urticaria, Rhinitis, allergic

## Abstract

**OBJECTIVES:**

Exposure to air pollutants is well established as being associated with the development and exacerbation of asthma. However, relatively few studies have examined the relationship between fine particulate matter (PM_2.5_) exposure and the risk of urticaria and allergic rhinitis (AR). Therefore, this study aimed to investigate the associations between long-term PM_2.5_ exposure and the risks of urticaria and AR.

**METHODS:**

We analyzed data from the Korean National Health Insurance Service–National Sample Cohort spanning 2002 to 2019. The cohort included individuals enrolled from birth and followed until the onset of urticaria or AR. Annual PM_2.5_ exposure was estimated using validated machine learning–based prediction models with high spatial resolution across mainland Korea. At the initial healthcare visit, urticaria and AR were identified based on primary diagnostic codes from the 10th revision of the International Classification of Diseases. Cox proportional hazards models with time-varying exposures and confounders were used to estimate hazard ratios (HRs) and 95% confidence intervals (CIs) for the risks associated with a 5 μg/m^3^ increase in annual PM_2.5_ concentration.

**RESULTS:**

During follow-up, 364,227 individuals aged 0–18 years at enrollment were included, among whom 151,508 developed urticaria and 258,041 developed AR. The mean PM_2.5_ concentration at baseline was 29.6 μg/m^3^ (standard deviation, 3.1). The HR per 5 μg/m^3^ increase in annual PM_2.5_ concentration was 1.02 (95% CI, 1.01 to 1.03) for urticaria and 1.06 (95% CI, 1.05 to 1.07) for AR.

**CONCLUSIONS:**

In this nationwide cohort, long-term exposure to PM_2.5_ was associated with the incidence of urticaria and AR. These findings provide important evidence for strategies aimed at preventing urticaria and AR in children.

## GRAPHICAL ABSTRACT

[Fig f5-epih-48-e2026020]

## Key Message

Long-term exposure to PM_2.5_ was associated with increased risks of urticaria and allergic rhinitis in Korean children and adolescents. In this nationwide cohort study, higher PM_2.5_ exposure was consistently linked to a greater risk of developing allergic diseases. These findings underscore the importance of air quality improvement for the prevention of pediatric allergic diseases.

## INTRODUCTION

Urticaria and allergic rhinitis (AR) affect approximately 25% and 30% of the global population, respectively, and represent substantial public health burdens [1]. Urticaria is characterized by the development of wheals (hives), angioedema, or both and may arise from allergic reactions to triggers such as food, drugs, pollen, and house dust [2]. However, in most cases of acute spontaneous urticaria, the underlying cause remains unknown, and its pathogenesis is not fully understood [3]. AR is an inflammatory condition of the nasal mucosa triggered by aeroallergens [4]. Not all sensitized children develop AR, indicating that additional factors influence disease expression. Allergic diseases, including urticaria and AR, are complex conditions involving interactions between genetic and environmental factors [5].

Air pollutants represent persistent environmental hazards that may trigger immune responses and exacerbate allergic diseases. The prevalence of allergic diseases has increased markedly over the past 2 decades, particularly in low-income and middle-income countries [1]. During this period, environmental changes—including industrialization, urbanization, and climate change—have intensified, contributing to increased population exposure to elevated levels of fine particulate matter (PM_2.5_; >35 μg/m^3^) [6]. Previous study has reported associations between elevated particulate matter levels and the development of skin diseases, potentially mediated through oxidative stress and inflammatory cytokine pathways [7]. A Chinese epidemiological study reported a significant association between PM_2.5_ exposure and the prevalence of urticaria in adolescents [8]. In contrast, some studies have reported minimal or no association between AR and air pollution across age groups [9,10]. In children with asthma, short-term exposure has been associated with nasal allergic inflammation [11], and in adults, short-term PM_2.5_ exposure has been linked to an increased risk of AR [12]. More recently, long-term PM_2.5_ exposure has been associated with the prevalence of allergic diseases among Chinese adults [13].

Therefore, this study aimed to conduct a comprehensive nationwide cohort analysis to investigate the association between long-term PM_2.5_ exposure during childhood and the risk of developing urticaria and AR. This analysis utilized a machine learning–based air pollution prediction model with a spatial resolution of 1 km^2^.

## MATERIALS AND METHODS

### Study area and population

We examined longitudinal data from 1,137,861 beneficiaries enrolled in the National Health Insurance Service–National Sample Cohort (NHIS–NSC). This cohort represents a 2.2% sample of the total eligible population in Korea between 2002 and 2019 [14]. The NHIS covers approximately 97% of the Korean population, excluding individuals who are ineligible because of death or emigration. Each year, the cohort was refreshed by adding a representative sample of newborns from 82 strata, defined by sex and parental income level, at a sampling rate of 2.2%. This study included participants from all 226 mainland districts (*si/gun/gu*) in Korea, with an average district size of 435.6 km^2^. The cohort comprises 4 databases containing information on insurance eligibility, including sex, year of birth and death, income status, and residential area. It also includes medical treatment data, such as diagnoses, prescriptions, and general health examination records [14]. In this study, the insurance eligibility and medical treatment databases were used to identify the study population, socioeconomic status, and onset of urticaria and AR.

**Figure 1.**
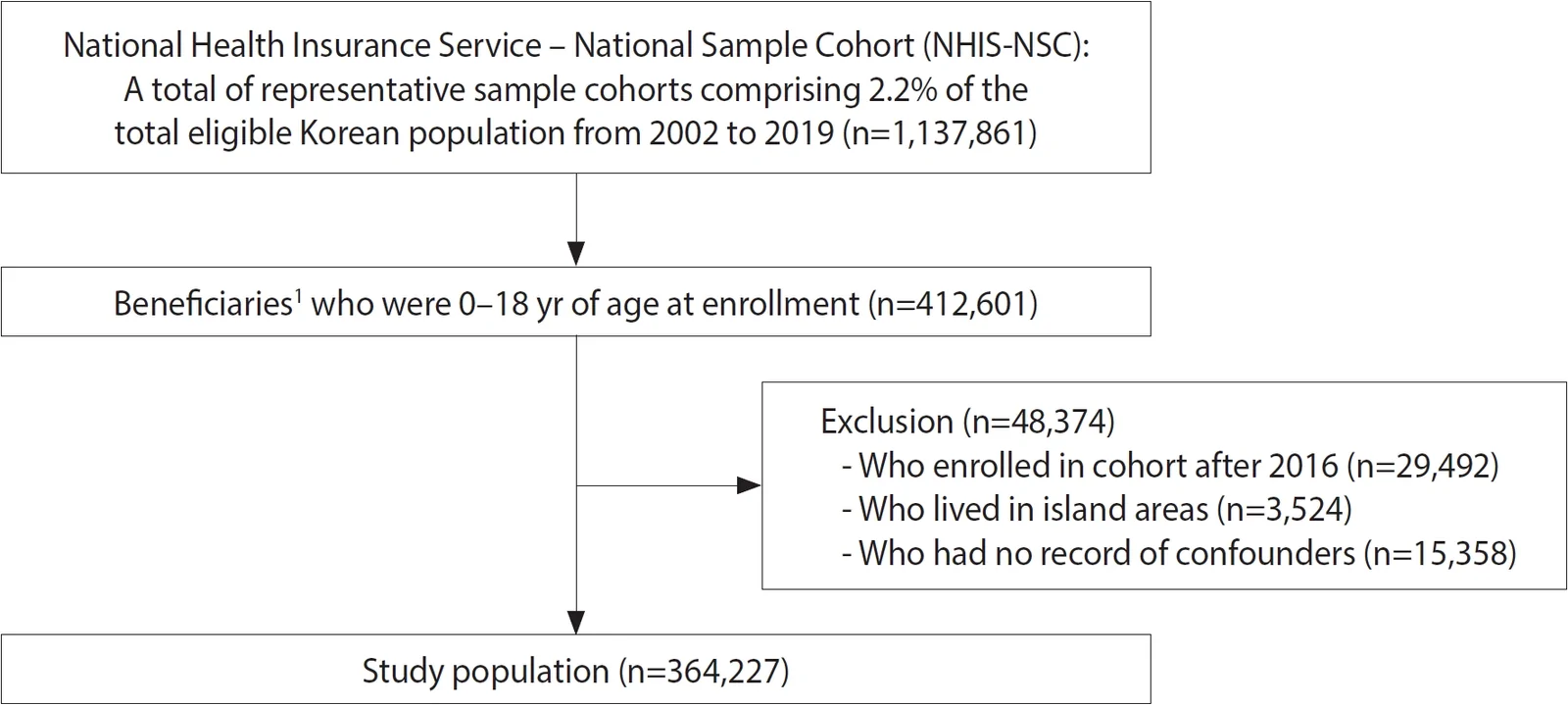
Flow diagram showing the selection process for the study population. 1The cohort was recruited annually by a representative sample of newborns, sampled across 82 strata (sex, combined with 41 for parents’ income levels) from NHIS-NSC.

Among 412,601 individuals aged 0–18 years at enrollment, 29,492 individuals who entered the cohort in or after 2016 were excluded to ensure at least 5 years of follow-up based on yearly updated data available through 2019. We also excluded 3,524 individuals residing on Jeju or Ulleung Island, where air pollution prediction values were unavailable, and 15,358 individuals with missing confounder data. Overall, 364,227 individuals were included and followed between 2002 and 2019 (Figure 1).

### Case definition

Medical data from primary diagnosis and treatment records in the medical treatment database were used to identify the onset of urticaria and AR. Disease onset was defined as the first healthcare visit, including outpatient visits and hospitalizations, at which the relevant primary diagnosis code was recorded. Diseases were identified according to the International Classification of Diseases, 10th revision (ICD-10). To comprehensively capture the burden of urticaria and AR across the full spectrum of disease severity, outcomes were defined using ICD-10–based diagnoses from all healthcare settings rather than being restricted to inpatient admissions. The specific ICD-10 codes used for each disease are described in the Supplementary Material 1.

### Air pollution data

Air pollution prediction data were obtained from the AiMS-CREATE network (Ai-Machine Learning and Statistics Collaborative Research Ensemble for Air Pollution, Temperature, and All Types of Environmental Exposures; https://www.datascience4health.com/). These predictions provided monthly ambient air pollutant concentrations at a spatial resolution of 1 km^2^ across all 226 districts in mainland Korea. Jeju and Ulleung Islands were excluded because of unstable prediction performance, potentially related to their distance from the nearest mainland area (Jeju, 80 km; Ulleung, 130 km). No PM_2.5_ concentration values were missing because modeled prediction data were used. The predictions were generated using validated machine learning models that showed strong performance for PM_2.5_ (cross-validated R^2^=0.87) [15].

Specifically, predictor variables from Google Earth Engine, the Socioeconomic Data and Applications Center, and other sources were used to predict PM_2.5_ concentrations. Eighty percent of monitoring stations were used for model training, and the remaining 20% were used for testing. Three machine learning models—random forest regression, gradient boosting, and neural networks—were trained using 10-fold cross-validation. Their performance, along with that of an ensemble model, was evaluated using R^2^ and root mean square error. The trained models were then applied to estimate monthly PM_2.5_ concentrations on a 1 km×1 km grid from 2002 to 2020. These gridded estimates were used to calculate annual average PM_2.5_ concentrations for each district (*si/gun/gu*), the smallest geospatial unit available in the NHIS–NSC database. Accordingly, previous studies using NHIS–NSC data have been conducted at the district level. Annual mean PM_2.5_ levels from the year preceding each follow-up year were assigned to participants based on their residential districts and updated annually. Additional details on the prediction model are provided in the Supplementary Material 2.

### Community-level indicators

To account for community-level socioeconomic status, data from the Korea Community Health Survey (KCHS) were used. Since 2008, the Korea Centers for Disease Control and Prevention (currently the Korea Disease Control and Prevention Agency) has conducted the KCHS, an annual nationwide cross-sectional health survey administered at the individual level [16]. The survey provides diverse health indicators that enable estimation of disease prevalence and regional health trends. For this study, we used a database of district-level health outcomes and determinants derived from KCHS data from 2008 to 2018. Six community-level socioeconomic and environmental indicators from this database were included in the model. Detailed model specifications are provided in the Statistical Analysis section. Covering 226 districts, the dataset underwent linear interpolation to address missing values for specific periods, assuming a linear relationship between observed values.

### Statistical analysis

Participants were followed from enrollment until the first onset of the outcome of interest, the end of the study period, or death, whichever occurred first. A single total cohort was defined at enrollment, and separate Cox regression models were fitted for urticaria and AR as independent outcomes, using the same underlying cohort. For each outcome-specific analysis, follow-up time was calculated from cohort entry to the first diagnosis of the respective condition, censoring, or the end of the study period. Cox proportional hazards models with time-varying exposures and confounders were used to estimate hazard ratios (HRs) and 95% confidence intervals (CIs) for the associations between long-term PM_2.5_ exposure and the risks of urticaria and AR [17]. The models were adjusted for potential time-varying confounders, including age (in 10-year units), individual income status, and community-level socioeconomic status indicators from the KCHS. These covariates were selected based on prior cohort studies and their potential to influence both PM_2.5_ exposure and the risks of urticaria and AR [16,18,19]. The community-level indicators included current smokers (%), number of hospital beds per 1,000 population, annual rate of non-treatment for medically necessary services (%), percentage of the population living in developed areas, basic livelihood support recipients per 1,000 population, and park area per person (km^2^). These variables were selected to capture area-level differences in healthcare access, socioeconomic conditions, and environmental context that may affect disease occurrence and its association with PM_2.5_ exposure. In addition, indicator variables for residential region (Capital, Mid, and South) were included to account for potential residual confounding from spatial trends. Sex, the only time-invariant confounder, was also included in the main model. Further details and rationale for the covariates are provided in the Supplementary Material 3.

#### Subgroup analysis

To identify subgroups potentially more vulnerable to PM_2.5_ exposure, effect modification was explored by sex (male or female), income level (low: deciles 0–2, with decile 0 referring to medical aid recipients; middle: deciles 3–7; high: deciles 8–10), age at disease onset (0–5, 6–10, and ≥11 years), and urbanicity. Urbanicity was categorized as urban (Gyeonggi-do and metropolitan cities, including Seoul, Incheon, Daejeon, Sejong, Busan, Daegu, Gwangju, and Ulsan) or rural (all other areas). Gyeonggi-do, a province surrounding Seoul, was considered to have a level of urbanization comparable to that of metropolitan cities.

#### Sensitivity analysis

Sensitivity analyses were conducted to assess the robustness of the main findings under alternative outcome definitions, exposure windows, and model specifications, including different covariate adjustment strategies. First, the disease definition was expanded by including secondary diagnoses in addition to primary ICD-10 diagnoses (ICD-10 version 2019). A secondary diagnosis refers to a condition coexisting with the primary diagnosis during a healthcare visit, allowing broader disease definitions to be evaluated. Second, current-year, 1-year lag, and 2-year lag exposure periods, as well as moving averages of PM_2.5_ concentrations, were evaluated as alternative exposure windows based on previously described approaches [20-22]. Third, community-level socioeconomic indicators were excluded from the main model to focus on individual-level factors. Fourth, to apply a more stringent case definition, outcomes were restricted to diagnoses recorded in inpatient settings only. Fifth, to account for potential regional heterogeneity and unmeasured area-level factors, residential regions (Capital, Mid, and South) were included as random effects in the model. Finally, to explore potential effect modification of PM_2.5_ associations according to the presence and levels of other pollutants, nitrogen dioxide (NO_2_) and ozone (O_3_) were added to models that already included PM_2.5_. NO_2_ and O_3_ were selected because their prediction models had high R^2^ values (>0.8), supporting the validity of the corresponding effect estimates. This approach allowed us to explore whether the associations between PM_2.5_ and the onset of urticaria or AR were influenced by varying levels of NO_2_ and O_3_ exposure, thereby providing insight into the potential combined effects of these pollutants.

All statistical analyses were conducted using SAS version 9.4 (SAS Institute Inc., Cary, NC, USA).

### Ethics statement

This study was approved by the Institutional Review Board of Ewha Womans University Seoul Hospital (IRB No. SEUMC 2022-05-034). The requirement for informed consent was waived because the dataset was anonymized and contained no personally identifiable information.

## RESULTS

### Descriptive statistics

The cohort included 364,227 beneficiaries residing in 226 districts. Among them, 151,508 participants (41.6%) developed urticaria, and 258,041 (70.8%) were diagnosed with AR. Baseline characteristics of the study population are presented in Table 1. At enrollment, the mean PM_2.5_ concentration was 29.6 μg/m^3^ (standard deviation [SD], 3.1). The mean age of the cohort was 6.1 years, and 52.3% of participants were boys. The low-income group accounted for 8.0% of the cohort, and 48.8% resided in the capital region. Total follow-up time was 4,342,197 person-years for urticaria and 3,220,960 person-years for AR. The median follow-up time was 14 years for urticaria and 7 years for AR, reflecting differences in the timing of first diagnosis between the 2 outcomes (Table 2). Supplementary Material 4 shows temporal trends in disease incidence. Urticaria incidence increased gradually until approximately 2013 and then declined, whereas AR incidence was higher in the early study period and gradually decreased thereafter.

### Air pollution exposure

**Figure 2.**
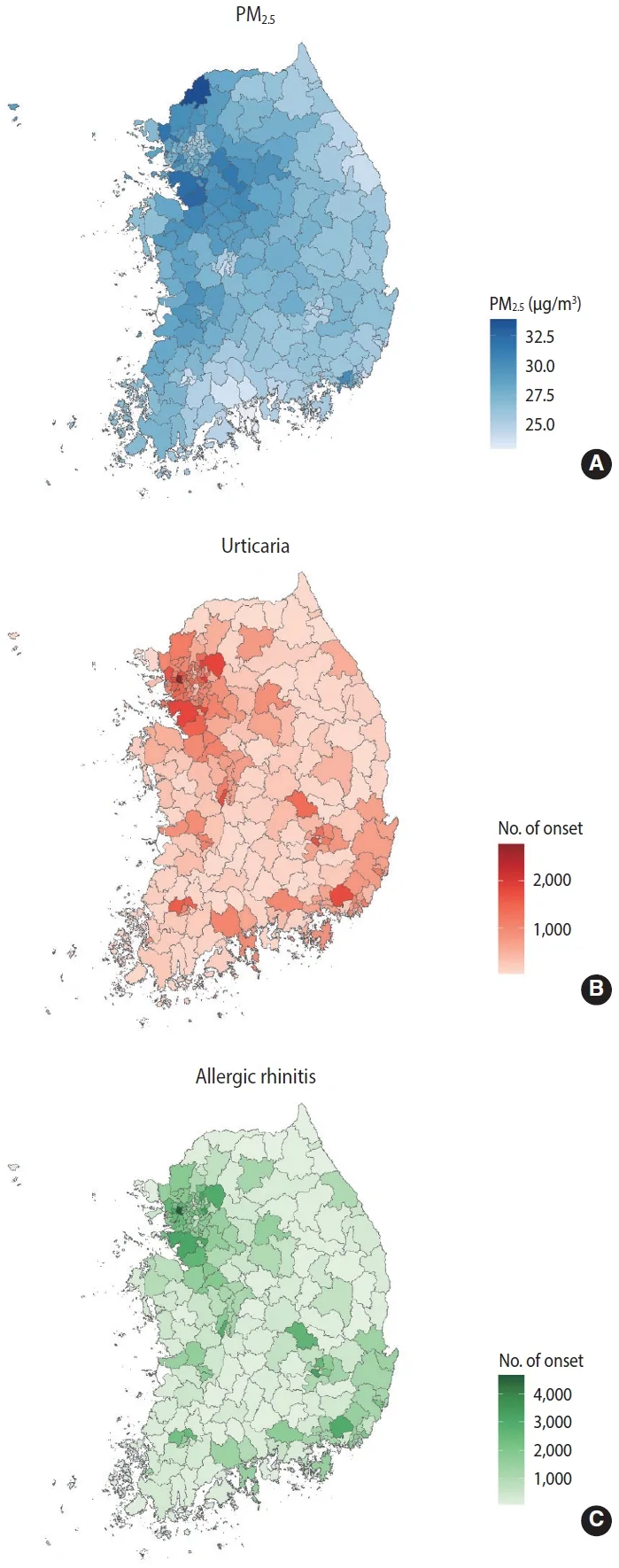
Nationwide distribution of fine particulate matter (PM_2.5_) concentrations and allergic disease occurrence across Korea. (A) Mean annual PM_2.5_ concentration (μg/m^3^). Occurrence of the first healthcare visit for (B) urticaria and (C) allergic rhinitis.

Across the study period, the mean±SD PM_2.5_ concentration was 29.6±3.1 μg/m^3^. The individual-level mean±SD PM_2.5_ exposure during follow-up was 27.2±3.1 μg/m^3^ for the urticaria analysis and 27.6±3.1 μg/m^3^ for the AR analysis, reflecting each participant’s average exposure from cohort entry until outcome occurrence or censoring (Table 2). PM_2.5_ levels were notably higher in metropolitan and western coastal areas, including Busan Metropolitan City (Figure 2A). First diagnoses of both diseases were recorded predominantly in metropolitan and capital areas, corresponding to areas with higher PM_2.5_ concentrations (Figure 2B and C). In addition, Supplementary Material 4 shows the temporal trend in PM_2.5_ concentrations over the study period. Annual mean PM_2.5_ levels declined consistently from 30.5 μg/m^3^ in 2002 to 23.4 μg/m^3^ in 2019, reflecting sustained efforts to reduce air pollution in Korea.

### Associations of exposure to fine particulate matter with disease risk

**Figure 3.**
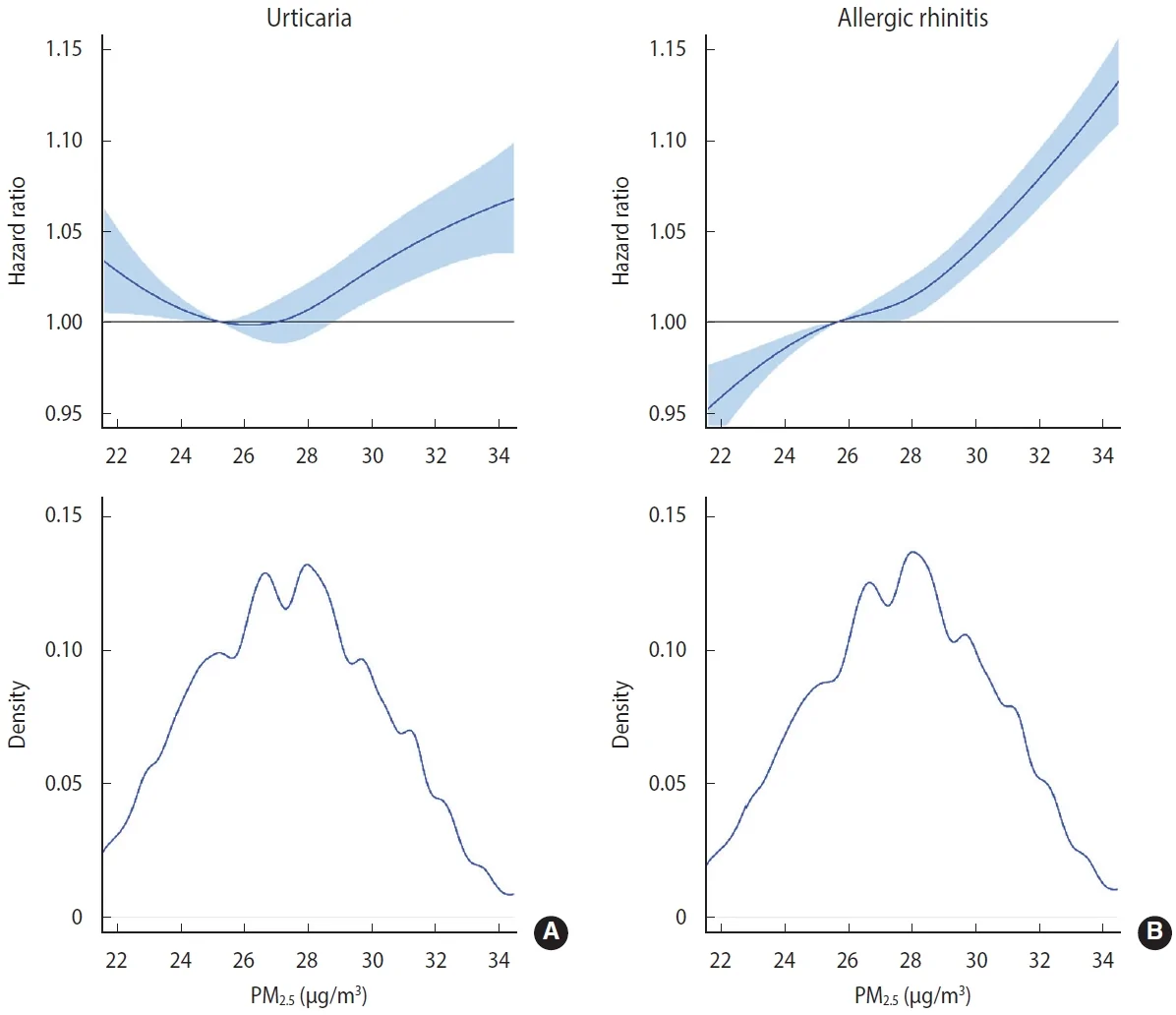
Concentration-response curves showing the association between long-term fine particulate matter (PM_2.5_) exposure and allergic diseases. Shaded areas represent 95% confidence intervals. Curves are shown for (A) urticaria and (B) allergic rhinitis.

In this cohort, AR showed a stronger positive association with long-term PM_2.5_ exposure than urticaria. The HR per 5 μg/m^3^ increase in PM_2.5_ concentration was 1.06 (95% CI, 1.05 to 1.07) for AR and 1.02 (95% CI, 1.01 to 1.03) for urticaria (Table 2). For AR, the association between PM_2.5_ concentration and HR appeared approximately linear, indicating higher risk at higher PM_2.5_ concentrations. In contrast, urticaria showed a J-shaped pattern, with an inflection point at the 25th percentile of PM_2.5_ exposure, indicating an increasing exposure-response relationship above this point (Figure 3).

**Figure 4.**
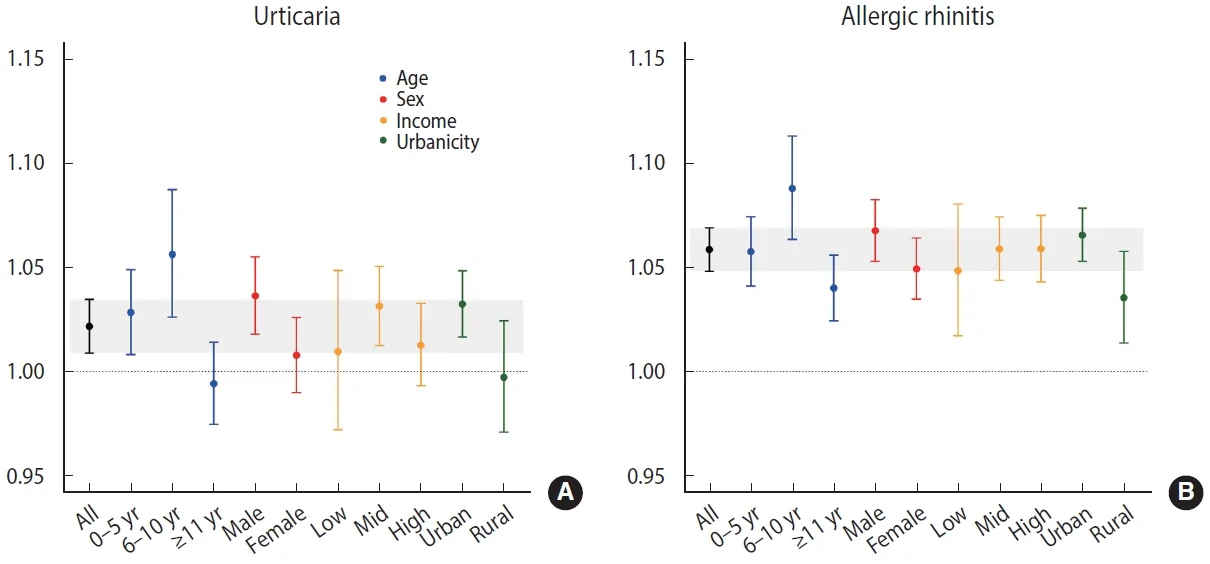
Identification of vulnerable subpopulations. Hazard ratios for each disease associated with a 5 μg/m^3^ increase in fine particulate matter (PM_2.5_) concentration are shown by subgroup (A) urticaria and (B) allergic rhinitis. Shading within the illustration represents the estimated main effects for the entire population. The blue line represents age groups based on age at disease onset. The red line represents sex-specific estimates. The yellow line represents income status, classified as low (deciles 0–2, including medical aid recipients), middle (deciles 3–7), or high (deciles 8–10). The green line represents residential urbanicity, classified as urban (Gyeonggi-do and metropolitan cities, including Seoul, Incheon, Daejeon, Sejong, Busan, Daegu, Gwangju, and Ulsan) or rural (all other areas).

In subgroup analyses, males appeared more susceptible than females to developing urticaria and AR after prolonged PM_2.5_ exposure (Figure 4). In analyses stratified by age at disease onset, the highest HRs were observed among individuals aged 6–10 years for both urticaria and AR (Figure 4). Differences by income level were not clearly significant. However, analyses by urbanicity showed higher HRs for both urticaria and AR among urban residents than among rural residents.

### Sensitivity analysis

The sensitivity analyses generally supported the main findings. The results remained robust when alternative PM_2.5_ exposure windows were used, secondary diagnoses were incorporated, community-level socioeconomic indicators were excluded, and residential areas were modeled as random effects. Adding another air pollutant to the model led to slight decreases in the estimates for both outcomes; however, these estimates did not differ substantially from the main findings. In contrast, restricting the outcome definition to hospitalization-only cases substantially reduced the number of events, resulting in limited statistical power and less stable estimates (Supplementary Material 5).

## DISCUSSION

This nationwide longitudinal cohort study found that long-term PM_2.5_ exposure was associated with increased incidence of urticaria and AR in children. Cox proportional hazards models with time-dependent exposure were used. This refined modeling approach enabled a more granular assessment of ambient air pollution than approaches based solely on fixed monitoring station data. Korea operates a universal healthcare system, and patient medical records are compiled within the NHIS. By including patients from diverse geographic areas across national subregions aligned with national ambient pollution mapping, this study accounted for temporal, spatial, and heterogeneous trends in air pollution across regions, particularly urban and rural areas.

Between 2006 and 2020, average annual PM_2.5_ concentrations ranged from 30 μg/m^3^ to 59 μg/m^3^ in China and from 9 μg/m^3^ to 15 μg/m^3^ in Japan [23-25]. In Korea, the mean PM_2.5_ concentration was 27.2 μg/m^3^ between 2002 and 2019, with higher levels typically observed in metropolitan and western coastal areas, potentially influenced by neighboring countries. Kim et al. [7] suggested that particulate matter can adhere to or penetrate the surface of the human epidermis, contributing to impaired skin homeostasis through skin cytotoxicity and systemic toxicity. A previous study has linked particulate matter–induced skin inflammation to oxidative stress and programmed cell death [7]. The present study found a positive association between long-term PM_2.5_ exposure and urticaria. It also showed a positive association between long-term PM_2.5_ exposure and AR. One study reported that exposure of nasal epithelial cells to PM_2.5_ reduces junction protein expression and increases the release of cytokines, including interleukin-8, TIMP metallopeptidase inhibitor 1, and thymic stromal lymphopoietin, which have proinflammatory properties. This process disrupts barrier function and supports the hypothesis that susceptibility to rhinitis increases in areas with high PM_2.5_ levels [26].

Aeroallergens primarily consist of proteins derived from plants, animals, and fungi. PM_2.5_ can adhere to allergen surfaces and alter allergenic proteins, particularly those in fungal spores and pollen fragments [27]. Recent studies have shown that simultaneous exposure to aeroallergens and air pollutants may contribute to allergic sensitization and allergic disease [5]. A previous study reported that higher urbanization levels and proximity to heavily trafficked roads are risk factors for AR [28]. In the present study, the risks of urticaria and AR associated with PM_2.5_ exposure were slightly higher in urban environments than in rural environments. These epidemiological findings underscore the need for further research on changes in allergenicity resulting from interactions between air pollutants and aeroallergens.

In subgroup analyses of long-term PM_2.5_ exposure, individuals aged 0–5 years had a higher risk of AR than those aged ≥6 years. Li et al. [29] conducted a 2022 meta-analysis examining the risk of AR in relation to several air pollutants, including PM_2.5_, coarse particulate matter (PM_10_), NO_2_, SO_2_, O_3_, and carbon mono-oxide. Their findings indicated that children and adolescents may be more sensitive than adults to the effects of air pollution on AR. In addition, males had a higher risk than females for both urticaria and AR in relation to long-term PM_2.5_ exposure. However, studies in China have reported that the effects of long-term PM_2.5_ exposure on lung function were greater in girls than in boys [30], and that PM_2.5_ exposure was associated with a higher risk of respiratory tract infection and cardiovascular-related hospitalization among females than among males [31]. These differences may reflect variation in biological susceptibility to specific pollutants, as well as sex-related behavioral differences in air pollution exposure. Further research is needed to clarify the relationships among air pollutants, age, and sex.

A marked increase in the prevalence of AR has been observed among school-aged children, consistent with findings from several studies. In a single-center study, the incidence of urticaria among Korean children increased from 73 per 100,000 in 2011 to 187 per 100,000 in 2018, whereas the incidence among adults remained stable [32]. Another study reported that the prevalence of AR was 9.0% in infants, 20.2% in preschool children, and 27.6% in school-aged children [33]. In the present study, 41.6% of patients developed urticaria, whereas 70.8% developed AR. The relatively high prevalence observed in this study may be explained by several factors. First, the broad diagnostic criteria for urticaria and AR may have contributed to higher estimates. Diagnosis of urticaria and AR is primarily based on clinical evaluation of symptoms combined with the patient’s medical history. Because urticaria and AR are heterogeneous and may have transient clinical manifestations, diagnosis can be challenging, potentially leading to misclassification. In addition, prevalence was estimated using the first recorded ICD-10 code as the diagnosis in this study. The inclusion of a broad range of ICD-10 codes may therefore have contributed to overestimation of prevalence. Second, approximately 2.2% of the Korean population was included in this cohort. Individuals in this cohort may have been more likely to receive a diagnosis than those who were not included, which may have influenced the study findings.

This study has several limitations. First, annual PM_2.5_ averages were used as the exposure measure for the year of initial urticaria or AR diagnosis. This approach could imply that children born later in the year, such as in December, were exposed to the annual PM_2.5_ average for only a few months. To address this concern, a sensitivity analysis was performed using PM_2.5_ averages from the year preceding the onset of urticaria or AR as lagged exposure variables, and the results were consistent. Second, this study included only mainland residents and excluded residents of Jeju and Ulleung Islands because PM_2.5_ prediction data were limited. Third, PM_2.5_ exposure was assigned at the district (*si/gun/gu*) level because of data availability, which may have caused some exposure misclassification. However, because nearly half of the participants lived in metropolitan areas with smaller, more homogeneous districts, the impact of this bias is likely limited. Fourth, outcomes were defined using ICD-10 diagnostic codes from insurance claims data, which may not fully capture all clinical cases. Validation studies have shown that although ICD-10 codes can have high positive predictive value (PPV), their sensitivity may be limited. For example, a hospital-based study in Thailand reported variable sensitivity and relatively high PPV when ICD-10 codes alone were used [34]. Fifth, the cumulative incidence of urticaria and AR was relatively high over the long follow-up period. Nevertheless, because the primary objective was to examine the association between long-term PM_2.5_ exposure and the timing of first diagnosis, survival analysis using Cox proportional hazards models—with time-varying exposure, censoring, and tied event times handled using Efron’s method—was considered appropriate and methodologically robust. Finally, despite adjustment for multiple covariates, residual confounding may persist because of factors such as genetic susceptibility, comorbidities, seasonal variation, temperature, and humidity.

This study provides epidemiological evidence that long-term exposure to PM_2.5_ is associated with an increased risk of developing urticaria and AR. Urticaria and AR are important public health concerns because they disrupt daily life and are associated with increased healthcare costs. These findings on long-term PM_2.5_ exposure may provide foundational evidence for public health policies aimed at reducing environmental pollution and disease risk.

## Figures and Tables

**Figure f5-epih-48-e2026020:**
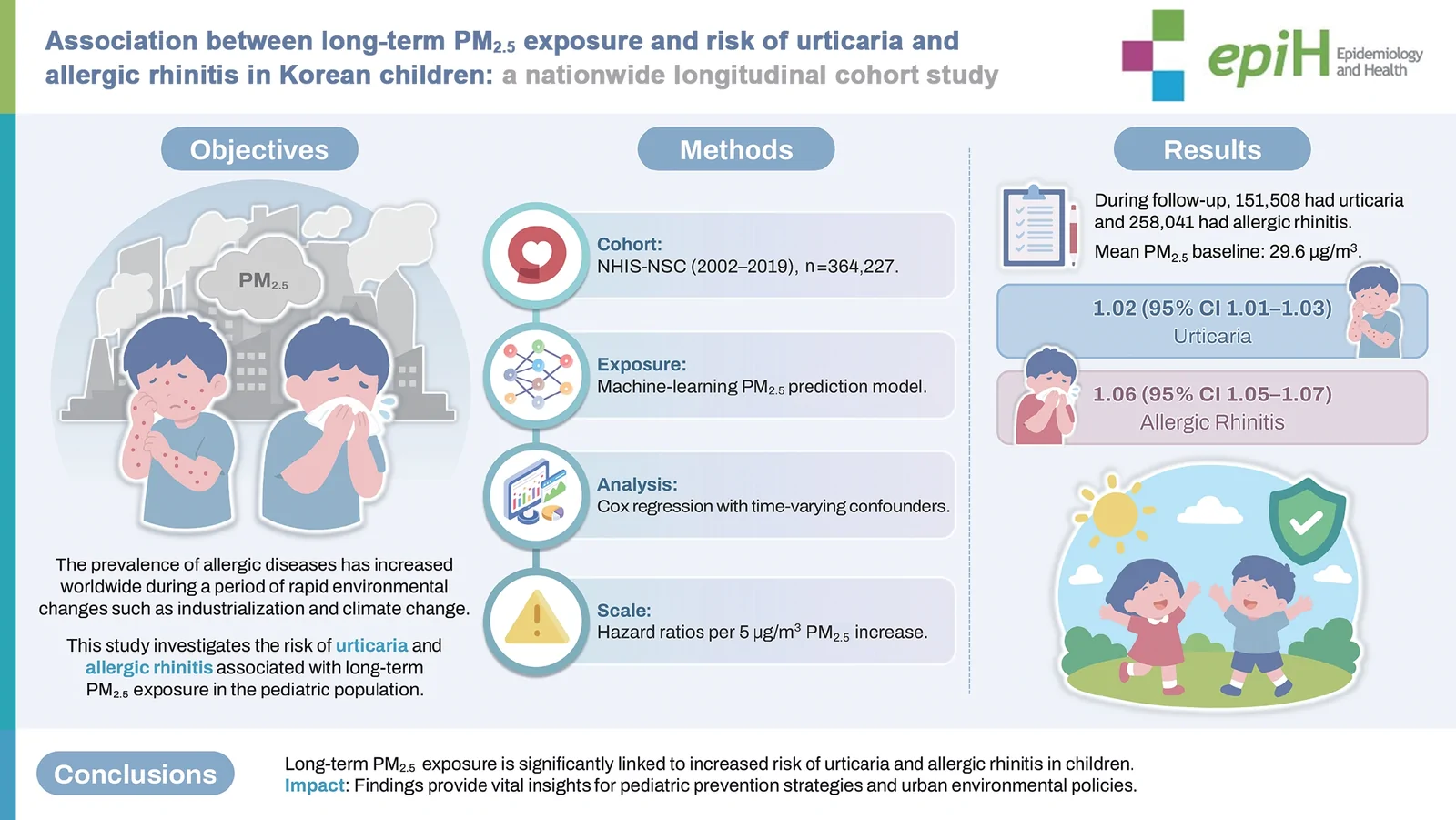


**Table 1.** Descriptive cohort characteristics for NHIS–NSC beneficiaries spanning 2002–2019 (n=364,227)

Characteristics	Mean±SD or n (%)^1^
Air pollutant concentration	
PM_2.5_ (μg/m^3^)	29.6±3.1
Potential confounders	
Age (yr)	
Mean±SD	6.1±6.1
0–9	249,755 (68.6)
10–19	114,472 (31.4)
Sex	
Male	190,520 (52.3)
Female	173,707 (47.7)
Income status	
Low (decile 0–2)^2^	29,040 (8.0)
Mid (decile 3–7)	195,064 (53.6)
High (decile 8–10)	140,123 (38.5)
Areas^3^	
Capital	177,612 (48.8)
Mid	48,520 (13.3)
South	138,095 (37.9)
Community-level indicators	
Current smokers (%)	25.6±2.6
No. of hospital beds per 1,000 population	10.4±5.1
Annual rate of non-treatment for medically necessary services (%)	13.0±4.1
Percentage of the population living in developed areas	90.0±18.2
Basic livelihood support recipients per 1,000 population	32.7±17.2
Park area per person (km^2^)	18.5±22.3

NHIS–NSC, National Health Insurance Service–National Sample Cohort; SD, standard deviation; PM2.5, fine particulate matter.

1 Descriptive characteristics were counted at enrollment for the entire cohort and at onset for patients with allergic diseases.

2 Decile 0 of income refers to recipients of medical aid.

3 Areas were divided by their approximate latitudes: Capital (Seoul, Incheon, and Gyeonggi), Mid (Daejeon, Sejong, Chungcheong, and Gangwon), and South (Busan, Daegu, Gwangju, Ulsan, Gyeongsang, and Jeolla); missing counts are for Jeju Island where air pollution is not projected.

**Table 2.** Associations between long-term PM_2.5_ concentration and first hospital visit for patients with allergic diseases

Variables	Urticaria	AR
No. of onsets	151,508	258,041
Total person-years	4,342,197	3,220,960
Median follow-up (yr)	14	7
Average PM_2.5_ concentration (μg/m^3^)	27.2±3.1	27.6±3.1
HR (95% CI) per 5 μg/m^3^ PM_2.5_	1.02 (1.01, 1.03)	1.06 (1.05, 1.07)

PM_2.5_, fine particulate matter; AR, allergic rhinitis; HR, hazard ratio; CI, confidence interval.

## Data Availability

This study used NHIS-NSC data (NHIS-2023-2-168) from the National Health Insurance Service (NHIS).

## Supplementary materials

Supplementary Material 1. Disease Definition

Supplementary Material 2. Air Pollution Prediction Model Design

Supplementary Material 3. Covariates Details

Supplementary Material 4. Temporal trends in annual mean PM_2.5_ concentrations and annual counts of incident urticaria and allergic rhinitis, 2002–2019.

Supplementary Material 5. Sensitivity analysis of the association between PM2.5 and onset of diseases.
