# Supplementary material for: Association between long-term PM2.5 exposure and risk of urticaria and allergic rhinitis in Korean children: a nationwide longitudinal cohort study
Source: Epidemiol Health. 2026 May 7;48:e2026020. doi: 10.4178/epih.e2026020 (PMC13503059; doi:10.4178/epih.e2026020)
Supplement: Supplementary file 1 [file epih-48-e2026020-Supplementary-1.docx]

**Supplementary Materials**

**Supplementary Material 1. Disease Definition**

We opted to include urticaria and allergic rhinitis in our study by referencing the International Classification of Diseases, 10th Revision (ICD-10). The selection of specific diseases was guided by the World Health Organization (WHO), which allowed us to identify the relevant ICD-10 codes for each condition.

| Disease | ICD-10 | | No. of patients (%) |
| --- | --- | --- | --- |
| Total cohort | - | | 364,227 (100.0) |
| Urticaria | L50.0 | Allergic urticaria | 151,508 (41.6) |
|  | L50.1 | Idiopathic urticaria |  |
|  | L50.3 | Dermatographic urticaria |  |
|  | L50.4 | Vibratory urticaria |  |
|  | L50.5 | Cholinergic uticaria |  |
|  | L50.6 | Contact urticaria |  |
|  | L50.8 | Other urticaria |  |
|  | L50.9 | Urticaria, unspecified |  |
| Allergic Rhinitis  (AR) | J30.1 | Allergic rhinitis due to pollen | 258,041 (70.8) |
|  | J30.2 | Other seasonal allergic rhinitis |  |
|  | J30.3 | Other allergic rhinitis |  |
|  | J30.4 | Allergic rhinitis, unspecified |  |
