# Supplementary material for: Association between long-term PM2.5 exposure and risk of urticaria and allergic rhinitis in Korean children: a nationwide longitudinal cohort study
Source: Epidemiol Health. 2026 May 7;48:e2026020. doi: 10.4178/epih.e2026020 (PMC13503059; doi:10.4178/epih.e2026020)
Supplement: Supplementary file 2 [file epih-48-e2026020-Supplementary-2.docx]

**Supplementary Materials**

**Supplementary Material 2. Air Pollution Prediction Model Design**

Air pollution prediction model was developed by AiMS-CREATE network where all authors are included. For the full process and result of prediction model, you could find more details in here: <https://papers.ssrn.com/sol3/papers.cfm?abstract_id=4280944>

*2.1 Modeling procedures*

Predictor variables collected from the google earth engine (GEE), Socioeconomic Data and Applications Center (SEDAC), regional socioeconomic database, and others were used as input variables, and PM_2.5_ concentration was predicted as the outcome value. Missing values in the predictor variables were replaced with values from the imputation. Randomly selected 80% of the monitoring stations were used to train the model, and the remaining 20% of the monitoring stations were used to test the model performance. Each of the three machine learning models were trained with 10-fold cross-validation (CV) in the training set to avoid overfitting. With these trained models, we checked the model performance of three machine learning models and simple averages among models (ensemble model) in the test set using R^2^ and root mean squared error (RMSE). Finally, we predicted monthly averages of air pollutant with a 1 km × 1 km grid during 2002–2020 using the three machine learning-based models (namely random forest, light gradient boosting, and neural network) and ensemble models.

*2.2 Ensemble model*

To aggregate the results, we calculated the simple averages of each machine learning estimation.

$$\hat{Y}_{SAij}= \frac{\hat{Y}_{RFij}+ \hat{Y}_{GBij}+ \hat{Y}_{NNij}}{3}$$

$\hat{Y}_{RFij}$, $\hat{Y}_{GBij}$, 𝑎𝑛𝑑 $\hat{Y}_{NNij}$ are PM_2.5_ estimations from the random forest, light gradient boosting, and neural network, respectively, at location i at time j; 𝑌_𝑆𝐴𝑖𝑗_ is the simple average estimation derived by averaging the three estimations at location i at time j. We also trained a generalized additive model (GAM) to consider the geographical variation of each of the three machine-learning estimations.

**Reference**

Park J, Kang C, Min J, et al. Association of long-term exposure to air pollution with chronic sleep deprivation in South Korea: A community-level longitudinal study, 2008-2018. Environ Res. 2023;228:115812. doi:10.1016/j.envres.2023.115812

Epstein LH, Raja S, Gold SS, Paluch RA, Pak Y, Roemmich JN. Reducing sedentary behavior: the relationship between park area and the physical activity of youth. Psychol Sci. 2006;17(8):654-659. doi:10.1111/j.1467-9280.2006.01761.x

Brown G, Schebella MF, Weber DJL, planning u: Using participatory GIS to measure physical activity and urban park benefits. Landscape and urban planning 2014, 121:34-44.
