# Supplementary material for: Association between long-term PM2.5 exposure and risk of urticaria and allergic rhinitis in Korean children: a nationwide longitudinal cohort study
Source: Epidemiol Health. 2026 May 7;48:e2026020. doi: 10.4178/epih.e2026020 (PMC13503059; doi:10.4178/epih.e2026020)
Supplement: Supplementary file 3 [file epih-48-e2026020-Supplementary-3.docx]

**Supplementary Materials**

**Supplementary Material 3. Covariates Details**

*3.1 Income status*

Income status was categorized into three ranges based on the decile of insurance premiums paid. These ranges are as follows:

- Low Income: Decile 0–2, where decile 0 represents individuals receiving medical aid.
- Mid Income: Decile 3–7.
- High Income: Decile 8–10.

The decile of insurance premiums serves as a proxy indicator for identifying different income levels. By utilizing information about insurance premiums paid by households, both employed beneficiaries and local beneficiaries are sorted into their respective deciles. This decile assignment is then applied to all individuals holding the same health insurance card.

The absence of insurance premium data pertains to beneficiaries who are exempt from individual insurance premium charges. This exemption includes individuals like professional soldiers and members of shipping unions. Moreover, data may be absent for beneficiaries who receive bills retrospectively due to factors such as extended periods of leave.

*3.2 Areas*

We divided Korea into three regions considering the latitude and proximity to the capital, Seoul.

- Capital: Seoul, Incheon, Gyeonggi.
- Mid: Decile Daejeon, Sejong, Chungcheong, Gangwon.
- South: Busan, Daegu, Gwangju, Ulsan, Gyeongsang, Jeolla.

*3.3 Community-level indicators*

- Current smokers (%):
  This indicator refers to the age-standardized percentage of people who have smoked more than 5 packs (100 cigarettes) in their lifetime who currently smoke ("every day" or "sometimes"). Although we could not address maternal smoking due to limitations in the NHIS-NSC data, additional adjustments were made based on the assumption that smoking rates in residential areas could potentially influence the incidence of urticaria and AR through environmental sanitation factors such as secondhand smoke.
- Number of hospital beds per 1,000 population:
  This indicator represents the total number of beds in medical institutions as defined in Article 3 of the Medical Service Act per 1,000 population. It is calculated by (total number of beds ÷ resident population) × 1,000. This variable reflects the healthcare infrastructure and accessibility in an area. Areas with fewer hospital beds may face challenges in providing timely and adequate healthcare services, potentially affecting the timely diagnosis of urticaria and AR cases.
- Annual untreated rate of medically necessary services (%):
  This indicator refers to the percentage of those who thought they needed medical treatment at hospitals and clinics (excluding dental) but did not receive treatment in the past year. High untreated rates may suggest potential delays in seeking or receiving medical care, and would potentially affect the diagnosis of urticaria and AR cases in this study.
- % of population living in developed areas:
  This indicator reflects the ratio of the population living in urban areas to the total population. Urban areas, in this context, are specifically defined as 'eup' and 'dong,' which are at a lower administrative level compared to districts (si/gun/gu). A higher percentage of residents in developed areas indicates that more people have enhanced access to healthcare, educational opportunities, and economic prospects. It can effectively serve as a proxy for socioeconomic status, which is a well-known factor influencing health outcomes, including both the incidence and management of conditions like urticaria and AR.
- Basic livelihood support recipients per 1,000 population:
  This indicator refers to the number of people receiving benefits under the National Basic Livelihood Security Act per 1,000 population. This measure of socioeconomic vulnerability. Higher numbers suggest a greater portion of the population relying on social assistance, which can indicate health inequities and disparities in access to healthcare.
- Park area per person (km^2^):
  The indicator refers to the total geographical size (km^2^) of all the parks per resident in each region. Previous studies have clarified that greater access to parks and the size of parks are positively associated with a high level of physical activity and a decrease in sedentary behaviors (Epstein et al., 2006; Brown et al., 2014). Conversely, it may also lead to increased exposure to outdoor air pollution, potentially raising the risk of health issues. Therefore, we hypothesized that this variable could influence the association between PM2.5 exposure and the onset of diseases.
