# Supplementary material for: Association between long-term PM2.5 exposure and risk of urticaria and allergic rhinitis in Korean children: a nationwide longitudinal cohort study
Source: Epidemiol Health. 2026 May 7;48:e2026020. doi: 10.4178/epih.e2026020 (PMC13503059; doi:10.4178/epih.e2026020)
Supplement: Supplementary file 4 [file epih-48-e2026020-Supplementary-4.docx]

**Supplementary Materials**

**Supplementary Material 4. Temporal trends in annual mean PM_2.5_ concentrations and annual counts of incident urticaria and allergic rhinitis, 2002–2019.**
