# Supplementary material for: Association between long-term PM2.5 exposure and risk of urticaria and allergic rhinitis in Korean children: a nationwide longitudinal cohort study
Source: Epidemiol Health. 2026 May 7;48:e2026020. doi: 10.4178/epih.e2026020 (PMC13503059; doi:10.4178/epih.e2026020)
Supplement: Supplementary file 5 [file epih-48-e2026020-Supplementary-5.docx]

**Supplementary Materials**

**Supplementary Material 5. Sensitivity analysis of the association between PM_2.5_ and onset of diseases.**

|  | Urticaria | AR |
| --- | --- | --- |
| Lag 0 (current year) | 1.02 (1.01, 1.04) | 1.08 (1.07, 1.09) |
| Lag 1 | 1.02 (1.01, 1.03) | 1.06 (1.05, 1.07) |
| Lag 2 | 1.02 (1.01, 1.03) | 1.04 (1.03, 1.05) |
| Lag 0–1 | 1.03 (1.01, 1.04) | 1.08 (1.07, 1.09) |
| Lag 0–2 | 1.03 (1.02, 1.05) | 1.07 (1.06, 1.08) |
| Including secondary diagnoses | 1.01 (0.99, 1.03) | 1.04 (1.03, 1.04) |
| Excluding regional indicators | 1.03 (1.02, 1.04) | 1.05 (1.04, 1.05) |
| Two-pollutant model (+NO_2_) | 0.99 (0.98, 1.01) | 1.03 (1.02, 1.04) |
| Two-pollutant model (+O_3_) | 1.01 (1.00, 1.02) | 1.05 (1.04, 1.06) |
| Region as a random effect | 1.02 (1.01, 1.04) | 1.06 (1.05, 1.07) |
| Hospitalization-only cases | 0.88 (0.73, 1.06) | 1.07 (0.86, 1.32) |

Values are HR (95% CI) per 5 µg/m^3^ increase in PM_2.5_.

Abbreviations: CI, confidence interval; HR, hazard ratio; AR, Allergic Rhinitis.
